# Alcohol-related psychiatric inpatient admissions in Ireland – characteristics, trends and factors associated with first and repeat admissions, 2015–2024

**DOI:** 10.1007/s11845-026-04420-0

**Published:** 2026-06-06

**Authors:** Anne Doyle, Antoinette Daly, Ena Lynn

**Affiliations:** Health Research Board, Grattan House, 67-72 Lower Mount Street, Dublin 2, D02 H638 Ireland

**Keywords:** Alcohol-related, Psychiatric, Repeat admissions

## Abstract

**Background:**

The association between alcohol use and poor mental health is well known. Although psychiatric services and supports are typically delivered in community settings, some individuals require specialist inpatient care for alcohol-related disorders. For many, multiple admissions may be required, referred to as the ‘Revolving Door’ (RD) phenomenon.

**Aims:**

This study examines alcohol-related admissions to inpatient psychiatric units during a 10-year period (2015 – 2024) using data from the Irish National Psychiatric Inpatient Reporting System (NPIRS). The study aims to compare characteristics of first admissions and re-admissions to examine factors associated with RD admissions.

**Methods:**

This study used retrospective, anonymised NPIRS data. Descriptive statistics were conducted, and statistical significance was assessed using Pearson *X*^2^ tests.

**Results:**

During the study period, 5.9% of all inpatient admissions into psychiatric units were alcohol related and almost two-thirds were readmissions (63.8%). Both first and readmissions were predominantly single, male, employed and aged between 45 – 64 years with a diagnosis of alcohol dependency. Readmissions were significantly more likely to have a longer length of stay.

Key sex differences in alcohol-related admissions were noted. Male admissions were significantly more likely to be single and younger. Females were more likely to be older, private patients and have longer inpatient stays.

Rural areas accounted for a larger share of alcohol-related admissions.

**Conclusions:**

Despite accounting for less than 6% of all psychiatric admissions, alcohol-related psychiatric hospitalisations represent substantial healthcare costs. The findings from this study highlight the need for more targeted, sex-sensitive interventions to reduce the high level of readmissions.

**Supplementary Information:**

The online version contains supplementary material available at https://doi.org/10.1007/s11845-026-04420-0.

## Introduction

Alcohol use and mental health are inextricably linked but it is often unclear whether alcohol use contributes to poor mental health or is used as self-medication to manage symptoms. As a depressogen, alcohol alters brain chemistry, leading to mood disturbances and impaired decision-making [1–3]. This relationship has been well-documented globally, and in Ireland. For example, suicide was the third leading alcohol-related cause of death among those aged 15–49 years according to the 2019 Global Burden of Disease (GBD) study [4]. In Ireland, alcohol was detected in 44% of suicide toxicology reports [5], and one-third of self-harm hospital presentations involved alcohol [6]. Recognising this, the World Health Organization (WHO) has prioritised reducing alcohol use as an important suicide prevention measure [7].

Historically, alcohol-related disorders were treated in psychiatric units in Ireland, but the 2006 mental health policy document, A Vision for Change, recommended community-based treatment, resulting in a decline in inpatient admissions [8, 9]. However, accessing appropriate services for those with both mental health and substance use issues remained complex. The 2020 policy *Sharing the Vision* reversed this stance, calling for integrated dual-diagnosis care and specialised teams within mental health services, consistent with *Sláintecare* recommendations [10, 11].

Services and supports remain typically in community settings although there continue to be individuals who require specialist inpatient care in psychiatric units for their alcohol-related disorder, often repeatedly, referred to as the ‘Revolving Door’ (RD) phenomenon [12, 13]. RD admissions with any mental health condition, utilise disproportionate healthcare resources, with readmission rates for psychiatric conditions internationally ranging from 9% to 84% [12, 14, 15]. A 2007 study conducted in Ireland found 37% of admissions to psychiatric services were readmitted during a 5-year period and 34.1% of those with alcohol-related first admissions were re-admitted, with 6.3% being frequent users (≥ 4 readmissions) [16]. Repeat admissions are typically associated with psychotic disorder diagnosis [17], personality disorders [18] and drug and/or alcohol-related disorders [12, 13, 17–19]. RD admissions are also associated with younger patients, and lower socioeconomic status [15, 16, 20, 21].

This study examines alcohol-related admissions to inpatient psychiatric units in Ireland (2015–2024) using data from the National Psychiatric Inpatient Reporting System (NPIRS) [22]. We compare first and repeat admissions to identify factors associated with readmission including potential sex and urban-rural differences. Given that much existing data are aggregated, stratified analysis may reveal previously masked sex-specific risk factors [23]. Early identification of high-risk groups can inform targeted, cost-effective interventions.

### Ethical considerations

This study used retrospective, anonymised data for secondary analysis with no potential harm or risk to individuals. Trends and characteristics are reported at a group level. The NPIRS data processing is justified by the Health Research Board’s statutory authority per SI no. 279/1986 and Sect. 2(b) of SI No.305/2007 for public interest or in the exercise of official authority vested in the Health Research Board.

## Methods

### Data source and population

This retrospective observational study used anonymised data on admissions to inpatient units extracted from the NPIRS. NPIRS is a national epidemiological database which records data on patient admissions to, and discharges from, psychiatric hospitals and units on the Register of Approved Centres under the Mental Health Act 2001 throughout Ireland. This includes public Health Services Executive (HSE)/HSE-funded facilities, independent/private and private charitable units and the National Forensic Mental Health Services at the Central Mental Hospital.

Admissions represent events or episodes rather than persons and thus reflect the activity of inpatient services. All received data is submitted under pre-defined specifications and verified by the individual units. Data, including diagnosis, is recorded at source in the units and hospitals by clinical staff. This study includes secondary use of anonymised data on admission and discharges recorded by the NPIRS system over a 10-year period (2015–2024) with aggregated trends and characteristics reported at the group level. Diagnoses are categorised in accordance with the WHO’s International Classification of Diseases, 10th Revision (ICD-10) categories [24].

To examine the urban-rural burden of alcohol-related disorders admissions to psychiatric units, we analysed the county of residence of admissions using inferential statistics to explore any differences between urban and rural patients. For the purposes of this study, the counties of Ireland were classified as ‘rural’ if > 50% of the population of that county resides in rural locations based on the 2022 Census, or ‘urban’ if > 50% of the population reside in urban areas [25]. It should be noted however, that while psychiatric units provide services to both urban and rural populations, this analysis uses location as a proxy indicator for urban–rural distribution.

### Study variables

The NPIRS database records up to four admission and discharge diagnoses. In this study an alcohol-related disorder are disorders related to or resulting from abuse or misuse of alcohol, thus alcohol-related admissions are those defined as having a primary admission diagnosis of mental and behavioural disorders due to use of alcohol (F10) [24]. The analysis on readmissions consists of a study group comprising those with alcohol-related disorders who were re-admitted to psychiatric units, i.e. they had been admitted at least once before, and the comparison group was alcohol-related admissions who were admitted for the first time.

### Statistical analysis

Descriptive characteristics and differences in factors according to first admission vs. readmissions were analysed using cross–tabulation, and statistical significance was assessed using Pearson *χ*^2^ tests. Data analysis was conducted using IBM SPSS Statistics Version 29.0.2.0 (Armonk, NY: IBM Corp, 2023). For all analyses, a P value of less than 0.05 was considered to indicate statistical significance.

As the unit of analysis in this study was episodes, which may not correspond to unique individuals, repeat episodes could not be reliably linked. This meant that the dataset does not provide independent, person-level observations with sufficient covariate detail to support multivariable modelling and conducting regression analysis would violate the assumption of independence and yield misleading estimates. As our aim was to quantify associations between episode characteristics rather than to construct a predictive model or draw adjusted causal inferences and therefore, we have reported *x*^2^ values odds as the most appropriate and transparent analytic approach. These values offer a straightforward measure of association while remaining consistent with the structure and inherent limitations of the dataset.

## Results

### Study demographics

#### All alcohol-related admissions

Between 2015 and 2024, there were 163,715 inpatient admissions to adult units, of which 9,586 had an alcohol-related diagnosis, accounting for 5.9% of all inpatient admissions into psychiatric units over the 10-year period. Males accounted for a higher proportion among alcohol-related admissions (59.8%) compared to females (40.2%) (Table 1). Those aged 45–64 years represented 43.5% of all alcohol-related admissions, almost one-half were single (47.6%), and over one in every three alcohol-related admissions were discharged in less than one week (35.4%). Although representing a smaller proportion of admissions, 5.8% involved individuals between 16 and 25 years.

Where an individual’s occupation was known (75.6%), non-manual occupations had the highest percentage of alcohol-related admissions (12.8%), followed by lower professional occupations (9.5%) and 1.9% of alcohol-related admissions during the study period indicated as having no fixed abode.

Where the referral source was known (43.0%), 16.4% of alcohol-related admissions were referred by a GP or an out-of-hours GP/primary care service and 10.5% were referred by a hospital emergency department/assessment unit/liaison psychiatry service.

#### Characteristics of first, vs. readmission of those admitted with an alcohol-related diagnoses

Almost two-thirds of alcohol-related psychiatric admissions in the 10-year period were readmissions (63.8%) (Table 1). The demographic characteristics of both first ever admissions and those of repeat admissions did not differ substantially. For example, 62.6% of first admissions, and 58.2% of readmissions were male and 40.0% of first admissions and 45.4% of readmissions were aged 45–64 years. As one might expect, a higher percentage of those aged 65 years age and over were readmissions (12.8%) compared to first admissions (9.7%). Although the number of admissions that remained in psychiatric units for periods of 3 months – 12 months was low, there was a positive association between this longer length of stay among readmissions (*p* < .001). Where the referral source was known (43.0%), for both first and readmissions, ‘GP or out of hours GP service or primary care’ was the most common referral source. First admissions were more likely to be admitted to general hospital psychiatric units (46.5%) and readmissions were more likely to be admitted to independent/private and private charitable centres (52.9%). The most common secondary diagnoses was depressive disorders at 28.6% followed by other drug disorders at 26.5%. However, there was no significant association between presence of a secondary diagnosis and first or readmission.

#### Sex differences in characteristics of first, vs. readmission of those admitted with an alcohol-related diagnoses

Given the higher proportion of male admissions in the data, we stratified the data by sex to ensure one set of data was not masking potential factors for the other. This allowed us to examine sex differences for first and readmissions and indicated that similarities remained aligned with the total sample apart from marital status. Of the total sample, there was no significant difference between marital status on admission and order of admission (first or readmission) (*p* = .619). When analysed by sex this finding remained for females (*p* = .179), however, for males, there was a positive significant association between marital status and first, as well as readmissions, with male admissions more likely to be single. Sex differences were also observed for location or region of admission with a non-significant finding for the total sample for females whereas for males, there was a significant association between region and first and readmissions.

Further comparisons of men and women indicated that a higher proportion of men (for both first and readmissions) were unemployed (33.1% and 36.0% respectively) compared to women (20.9% and 22.9% respectively). Furthermore, 10.3% of women re-admitted were retired compared to 6.8% of men. Of readmissions, men were more likely to be single (55.3%) compared to 36.3% of women and women who were re-admitted were more likely to be married (38.2%) compared to 26.9% of men, although marital status was not statistically significant for women. A higher percentage of women were admitted to private facilities (61.5%) compared to men (42.5%) and women on average had a longer length of stay. Of women admitted for the first time, almost one-half were admitted for over one month (47.3% of women compared to 32.9% of men) and of readmissions, 32.6% of women had a length of stay for a period of one month or longer compared to 25.3% of men.

#### Geographical differences in characteristics of first, vs. readmission of those admitted with an alcohol-related diagnoses

The county location of alcohol-related admissions was used to determine whether those admitted resided in urban or rural locations. While 67.2% of admissions were considered urban residents, there was no significant urban-rural difference for order of admission overall or when examined by sex (Table 1). However, there was a significant urban-rural difference for disorder type (χ^2^ = 449.1; *p*<.001) with admissions for dependence syndrome higher among urban admissions than rural admissions. Significant urban-rural differences were also noted for age at admission whereby those being admitted from urban areas had an older age at admission (mean = 47.71) than rural admissions (mean = 45.17).

Further exploration based on the population of rural areas compared to urban areas, the rate per 100,000 of alcohol related disorders were higher in rural areas (191.3 per 100,000: 68.9 for first admissions and 122.4 for readmissions) compared with rates in urban areas (183.1 per 100,000: 66.2 for first admissions and 116.9 for repeat admissions). Rural counties Donegal and Sligo had substantially higher than the national rural average rates, at 465.6 alcohol-related psychiatric facility admissions per 100,000 of the population, the Sligo rate was 327.6 per 100,000.

**Table 1:** Characteristics of all, first, and readmissions for alcohol-related psychiatric hospital admissions, by sex, 2015–2024ⱡ

Characteristic	Total, *N* (%)	First ever admission	Readmissions	Total, *N* (%)	First ever admission, *N* (%)	Readmissions, *N* (%)	Total, *N* (%)	First ever admission, *N* (%)	Readmissions, *N* (%)
	All alcohol-related admissions			Male alcohol-related admissions			Female alcohol-related admissions		
*All*	9586 (5.9% of all admissions)	3471 (36.2)	6115 (63.8)	5732	2173 (37.9)	3559 (62.1)	3854 (67.2)	1298 (33.7)	2556 (66.3)
*Sex at birth*	**χ**^**2**^ **= 17.858;** ***p*<.001**								
*Male*	5732 (59.8)	2173 (62.6)	3559 (58.2)	-	-	-	-	-	-
*Female*	3854 (40.2)	1298 (37.4)	2556 (41.8)	-	-	-	-	-	-
*Age group at admission*	**χ**^**2**^ **= 92.653;** ***p*<.001**			**χ**^**2**^ **= 44.754;** ***p*<.001**			**χ**^**2**^ **= 40.682;** ***p*<.001**		
*Less than 25 years*	559 (5.8)	279 (8.0)	280 (4.6)	400 (7.0)	203 (9.3)	197 (5.5)	159 (4.1)	76 (5.9)	83 (3.2)
*25–44 years*	3739 (39.0)	1465 (42.2)	2274 (37.2)	2538 (44.3)	1003 (46.1)	1535 (43.1)	1201 (31.2)	462 (35.6)	739 (28.9)
*45–64 years*	4168 (43.5)	1390 (40.0)	2778 (45.4)	2256 (39.4)	791 (36.4)	1465 (41.2)	1912 (49.6)	599 (46.1)	1313 (51.4)
*65 + years*	1120 (11.7)	337 (9.7)	783 (12.8)	538 (9.4)	176 (8.1)	362 (10.2)	582 (15.1)	161 (12.4)	421 (16.5)
*Employment status*	**χ**^**2**^ **= 72.415;** ***p*<.001**			**χ**^**2**^ **= 44.257;** ***p*<.001**			**χ**^**2**^ **= 34.984;** ***p*<.001**		
*Employed*	3199 (33.4)	1079 (31.1)	2120 (34.7)	1914 (33.4)	674 (31.0)	1240 (34.8)	1285 (33.3)	405 (31.2)	880 (34.4)
*Unemployed*	2858 (29.8)	991 (28.6)	1867 (30.5)	2001 (34.9)	720 (33.1)	1281 (36.0)	857 (22.2)	271 (20.9)	586 (22.9)
*Retired*	731 (7.6)	227 (6.5)	504 (8.2)	368 (6.4)	126 (5.8)	242 (6.8)	363 (9.4)	101 (7.8)	262 (10.3)
*Student*	209 (2.2)	84 (2.4)	125 (2.0)	129 (2.3)	59 (2.7)	70 (2.0)	80 (2.1)	25 (1.9)	55 (2.2)
*Home duties*	250 (2.6)	80 (2.3)	170 (2.8)	< 5 (0.1)	< 5 (0.1)	< 5 (0.0)	246 (6.4)	77 (5.9)	169 (6.6)
*Other and unknown*	2339 (24.4)	1010 (29.1)	1329 (21.7)	1316 (23.0)	591 (27.2)	725 (20.4)	1023 (26.5)	419 (32.3)	604 (23.6)
*Marital status*	***p*** **=.619**			**χ**^**2**^ **= 10.955;** ***p*<.05**			***p*** **=.179**		
*Single*	4564 (47.6)	1667 (48.0)	2897 (47.4)	3119 (54.4)	1151 (53.0)	1968 (55.3)	1445 (37.5)	516 (39.8)	929 (36.3)
*Married*	3040 (31.7)	1106 (31.9)	1934 (31.6)	1593 (27.8)	636 (29.3)	957 (26.9)	1447 (37.5)	470 (36.2)	977 (38.2)
*Widowed*	331 (3.5)	109 (3.1)	222 (3.6)	137 (2.4)	45 (2.1)	92 (2.6)	194 (5.0)	64 (4.9)	130 (5.1)
*Divorced*	548 (6.7)	188 (5.4)	360 (5.9)	275 (4.8)	91 (4.2)	184 (5.2)	273 (7.1)	97 (7.5)	176 (6.9)
*Unspecified*	1103 (11.5)	401 (11.6)	702 (11.5)	608 (10.6)	250 (11.5)	358 (10.1)	495 (12.8)	151 (11.6)	344 (13.5)
*Length of stay*	**χ**^**2**^ **= 195.243;** ***p*<.001**			**χ**^**2**^ **= 96.318;** ***p*<.001**			**χ**^**2**^ **= 120.654;** ***p*<.001**		
*Under 1 week*	3283 (34.2)	1239 (35.7)	2044 (33.4)	2163 (37.7)	863 (39.7)	1300 (36.5)	1120 (29.1)	376 (29.0)	744 (29.1)
*1-< 2 weeks*	1776 (18.5)	530 (15.3)	1246 (20.4)	1123 (19.6)	355 (16.3)	768 (21.6)	653 (16.9)	175 (13.5)	478 (18.7)
*2-< 4 weeks*	1464 (15.3)	372 (10.7)	1092 (17.9)	831 (14.5)	240 (11.0)	591 (16.6)	633 (16.4)	132 (10.2)	501 (19.6)
*1-< 3 months*	2701 (28.2)	1205 (34.7)	1496 (24.5)	1408 (24.6)	639 (29.4)	769 (21.6)	1293 (33.5)	566 (43.6)	727 (28.4)
*3 months-< 1 year*	63 (0.7)	15 (0.4)	48 (0.8)	35 (0.6)	< 5 (0.2)	31 (0.9)	28 (0.7)	11 (0.8)	17 (0.7)
*Not discharged by year-end*	299 (3.1)	110 (3.2)	189 (3.1)	172 (3.0)	72 (3.3)	100 (2.8)	127 (3.3)	38 (2.9)	89 (3.5)
*Public or Private*	**χ**^**2**^ **= 49.359;** ***p*<.001**			**χ**^**2**^ **= 18.565;** ***p*<.001**			**χ**^**2**^ **= 23.05;** ***p*<.001**		
*Public*	4777 (49.8)	1895 (54.6)	2882 (47.1)	3294 (57.5)	1327 (61.1)	1967 (55.3)	1483 (38.5)	568 (43.8)	915 (35.8)
*Private*	4809 (50.2)	1576 (45.4)	3233 (52.9)	2438 (42.5)	846 (38.9)	1592 (44.7)	2371 (61.5)	730 (56.2)	1641 (64.2)
*Socio-economic group*	**χ**^**2**^ **= 38.859;** ***p*<.001**			**χ**^**2**^ **= 22.919;** ***p*<.05**			**χ**^**2**^ **= 33.966;** ***p*<.001**		
*Farmers*	121 (1.3)	41 (1.2)	80 (1.3)	112 (2.0)	39 (1.8)	73 (2.1)	9 (0.2)	< 5 (0.2)	7 (0.3)
*Agricultural Workers*	34 (0.4)	10 (0.3)	24 (0.4)	26 (0.5)	6 (0.3)	20 (0.6)	8 (0.2)	< 5 (0.3)	< 5 (0.2)
*Higher Professional*	466(4.9)	154 (4.4)	312 (5.1)	287 (5.0)	106 (4.9)	181 (5.1)	179 (4.6)	48 (3.7)	131 (5.1)
*Lower Professional*	907 (9.5)	281 (8.1)	626 (10.2)	381 (6.6)	127 (5.8)	254 (7.)	526 (13.6)	154 (11.9)	372 (14.6)
*Employers and Managers*	565 (5.9)	186 (5.4)	379 (6.2)	347 (6.1)	112 (5.2)	235 (6.6)	218 (5.7)	74 (5.7)	144 (5.6)
*Own Account Workers*	130 (1.4)	33 (1.0)	97 (1.6)	87 (1.5)	27 (1.2)	60 (1.7)	43 (1.1)	6 (0.5)	37 (1.4)
*Non-manual*	1228 (12.8)	436 (12.6)	792 (13.0)	519 (9.1)	200 (9.2)	319 (9.0)	709 (18.4)	236 (18.2)	473 (18.5)
*Manual Skilled*	729 (7.6)	269 (7.7)	460 (7.5)	657 (11.5)	256 (11.8)	401 (11.3)	72 (1.9)	13 (1.0)	59 (2.3)
*Semi-skilled*	392 (4.1)	143 (4.1)	249 (4.1)	264 (4.6)	96 (4.4)	168 (4.7)	128 (3.3)	47 (3.6)	81 (3.2)
*Unskilled*	429 (4.5)	140 (4.0)	289 (4.7)	354 (6.2)	118 (5.4)	236 (6.6)	75 (1.9)	22 (1.7)	53 (2.1)
*Unspecified*	4585 (47.8)	1778 (51.2)	2807 (45.9)	2698 (47.1)	1086 (50.0)	1612 (45.3)	1887 (49.0)	692 (53.3)	1195 (46.8)
*Referral source*	**χ**^**2**^ **= 93.172;** ***p*<.001**			**χ**^**2**^ **= 66.456;** ***p*<.001**			**χ**^**2**^ **= 33.2;** ***p*<.001**		
*ED/Assessment Unit/Liaison Psychiatry*	1005 (10.5)	404 (11.6)	601 (9.8)	700 (12.2)	287 (13.2)	413 (11.6)	305 (7.9)	117 (9.0)	188 (7.4)
*Other psychiatric hospital or unit*	88 (0.9)	32 (0.9)	56 (0.9)	54 (0.9)	22 (1.0)	32 (0.9)	34 (0.9)	10 (0.8)	24 (0.9)
*Other hospital/transferred from general hospital*	236 (2.5)	94 (2.7)	142 (2.3)	166 (2.9)	68 (3.1)	98 (2.8)	70 (1.8)	26 (2.0)	44 (1.7)
*Other hospital - not specified psychiatric or not psychiatric*	111 (1.2)	30 (0.9)	81 (1.3)	52 (0.9)	13 (0.6)	39 (1.1)	59 (1.5)	17 (1.3)	42 (1.6)
*Community residence or residential home or nursing home*	41 (0.4)	8 (0.2)	33 (0.5)	27 (0.5)	< 5 (0.2)	23 (0.6)	14 (0.4)	< 5 (0.3)	10 (0.4)
*Outpatient clinic or day hospital or day centre*	203 (2.1)	49 (1.4)	154 (2.5)	141 (2.5)	29 (1.3)	112 (3.1)	62 (1.6)	20 (1.5)	42 (1.6)
*GP or out of hours GP service or primary care*	1575 (16.4)	655 (18.9)	920 (15.0)	876 (15.3)	381 (17.5)	495 (13.9)	699 (18.1)	274 (21.1)	425 (16.6)
*Self-referral*	297 (3.1)	76 (2.2)	221 (3.6)	181 (3.2)	51 (2.3)	130 (3.7)	116 (3.0)	25 (1.9)	91 (3.6)
*Consultant*	80 (0.8)	20 (0.5)	60 (1.0)	45 (0.8)	13 (0.6)	32 (0.9)	35 (0.9)	7 (0.5)	28 (1.1)
*Justice - Garda*,* prison*,* courts*	395 (4.1)	179 (5.2)	216 (3.5)	281 (4.9)	131 (6.0)	150 (4.2)	114 (3.0)	48 (3.7)	66 (2.6)
*Community Mental Health Team or sector team*	91 (0.9)	22 (0.6)	69 (1.1)	65 (1.1)	17 (0.8)	48 (1.3)	26 (0.7)	5 (0.4)	21 (0.8)
*Other referral source or unspecified/unknown*	5464 (57.0)	1902 (54.8)	3562 (58.3)	3144 (54.8)	1157 (53.2)	1987 (55.8)	2320 (60.2)	745 (57.4)	1575 (61.6)
*Secondary diagnosis*	***p*** **=.063**			***p*** **=.267**			***p*** **=.061**		
*Has a secondary diagnosis*	2091 (21.8)	721 (20.8)	1370 (22.4)	1322 (23.1)	484 (22.3)	838 (23.5)	769 (20.0)	237 (18.3)	532 (20.8)
*No secondary diagnosis*	7495 (78.2)	2750 (79.2)	4745 (77.6)	4410 (76.9)	1689 (77.7)	2721 (76.5)	3085 (80.0)	1061 (81.7)	2024 (79.2)
*Hospital Type*	**χ**^**2**^ **= 60.286;** ***p*<.001**			**χ**^**2**^ **= 25.174;** ***p*<.001**			**χ**^**2**^ **= 26.952;** ***p*<.001**		
*General hospital psychiatric units*	3962 (41.3)	1613 (46.5)	2349 (38.4)	2775 (48.4)	1144 (52.6)	1631 (45.8)	1187 (30.8)	469 (36.1)	718 (28.1)
*Psychiatric hospitals/continuing care units*	815 (8.5)	282 (8.1)	533 (8.7)	519 (9.1)	183 (8.4)	336 (9.4)	296 (7.7)	99 (7.6)	197 (7.7)
*Independent/private and private charitable centres*	4809 (50.2)	1576 (45.4)	3233 (52.9)	2438 (42.5)	846 (38.9)	1592 (44.7)	2371 (61.5)	730 (56.2)	1641 (64.2)
*Region*	**χ**^**2**^ **= 26.51;** ***p*<.001**			***p*** **=.201**			**χ**^**2**^ **= 33.529;** ***p*<.001**		
*Dublin*	3365 (35.2)	1103 (31.9)	2262 (37.0)	1791 (83.0)	648 (30.0)	1143 (32.1)	1574 (40.9)	455 (35.1)	1119 (43.8)
*Rest of Leinster*	2163 (22.6)	810 (23.5)	1353 (22.1)	1273 (59.0)	507 (23.5)	766 (21.5)	890 (23.1)	303 (23.4)	587 (23.0)
*Munster*	2039 (21.3)	795 (23.0)	1244 (20.4)	1356 (62.8)	521 (24.1)	835 (23.5)	683 (17.8)	274 (21.2)	409 (16.0)
*Connacht/Ulster*	1995 (20.9)	745 (21.6)	1250 (20.5)	1295 (60.0)	482 (22.3)	813 (22.9)	700 (18.2)	263 (20.3)	437 (17.1)
*Urban-rural location* *	**χ**^**2**^ **= 0.019;** ***p*=.890**			**χ**^**2**^ **= 1.222;** ***p*=.269**			**χ**^**2**^ **= 0.784;** ***p*=.376**		
*Rural*	3121 (32.6)	1124 (32.6)	1997 (32.7)	1971 (34.6)	725 (33.6)	1246 (35.0)	1150 (29.9)	399 (30.8)	751 (29.4)
*Urban*	6441 (67.4)	2329 (67.4)	4112 (67.3)	3744 (65.5)	1433 (66.4)	2311 (65.0)	2697 (70.1)	896 (69.2)	1801 (70.6)

ⱡ Percentage totals may not add up to 100 due to rounding up/down

*Note non-residents (*n* = 24) were excluded from this analysis and therefore the total differs to others shown

### Alcohol-related disorder type

The most common type of alcohol-related disorder was dependence syndrome, accounting for almost one-half of alcohol-related disorder admissions (48.0%). Harmful use accounted for 6.7%, while acute intoxication accounted for 6.3%. Approximately one-third of first and of readmissions with an alcohol-related disorder did not have a specific clinical condition specified on admission. First and readmissions followed a very similar pattern to the overall sample (Table 2). Dependence syndrome was the most frequently reported diagnosis for both groups (44.8% of first admissions and 49.8% of readmissions). Harmful use of alcohol was more frequently reported among first admissions (7.5%) compared to readmissions (6.2%).

Sex differences followed a similar pattern with the exception of dependence syndrome, where a higher percentage of females had a diagnosis of dependence syndrome (51.5%) compared to the percentage of males with the same diagnosis (40.8%). Likewise for readmissions, 56.7% of females had a diagnosis of dependence syndrome at readmission compared to 44.8% of males.

The mean age of those admitted for the first time was 44.91 (median 44), readmissions were slightly older with a mean age of 48 (median 48). It is interesting to note that females were on average 5 years older on first admission (mean age 47.65 years) and on readmission (mean age 50.85 years) compared to males (mean age 43.28 and 45.96 years respectively) for all alcohol disorder types, with the exception of those presenting with a diagnosis of ‘other mental and behavioural disorders’ due to alcohol (Table 2).

**Table 2:** Number of first and readmission of alcohol-related admissions by sex, disorder type and age at admission, 2015–2024

	Total, *N* (%)	First admissions *n* (%)	Readmissions *n* (%)	Total *N* (%)	First admission *n* (%)	Readmissions *n* (%)	Total *N* (%)	First admission *n* (%)	Readmissions *n* (%)
	All alcohol-related admissions			Male alcohol-related admissions			Female alcohol-related admissions		
All	**9586**	**3471**	**6115**	**5732**	**2173 (37.9)**	**3559 (62.1)**	**3854**	**1298 (37.4)**	**2556 (41.8)**
Disorder type	**χ**^**2**^ **= 41.932; *p*<.001**			**χ**^**2**^ **= 24.737; *p*<.05**			**χ**^**2**^ **= 32.769;** ***p*<.001**		
Clinical condition not specified (F10)	3219 (33.6)	1224 (35.3)	1995 (32.6)	2101 (36.7)	814 (37.5)	1287 (36.2)	1118 (29.0)	410 (31.6)	708 (27.7)
Acute intoxication (F10.0)	605 (6.3)	207 (6.0)	398 (6.5)	403 (7.0)	152 (7.0)	251 (7.1)	202 (5.2)	55 (4.2)	147 (5.8)
Harmful use (F10.1)	642 (6.7)	262 (7.5)	380 (6.2)	405 (7.1)	176 (8.1)	229 (6.4)	237 (6.1)	86 (6.6)	151 (5.9)
Dependence syndrome (F10.2)	4598 (48.0)	1555 (44.8)	3043 (49.8)	2482 (43.3)	887 (40.8)	1595 (44.8)	2116 (54.9)	668 (51.5)	1448 (56.7)
Withdrawal state (F10.3)	100 (1.0)	40 (1.2)	60 (1.0)	59 (1.0)	26 (1.2)	33 (0.9)	41 (1.1)	14 (1.1)	27 (1.1)
Withdrawal state with delirium (F10.4)	33 (0.3)	16 (0.5)	17 (0.3)	22 (0.4)	11 (0.5)	11 (0.3)	11 (0.3)	5 (0.4)	6 (0.2)
Psychotic disorder (F10.5)	238 (2.5)	103 (3.0)	135 (2.2)	166 (2.9)	73 (3.4)	93 (2.6)	72 (1.9)	30 (2.3)	42 (1.6)
Amnesic syndrome (F10.6)	35 (0.4)	11 (0.3)	24 (0.4)	26 (0.5)	< 5 (0.2)	22 (0.6)	9 (0.2)	7 (0.5)	< 5 (0.1)
Residual and late onset psychotic disorder (F10.7)	21 (0.2)	10 (0.3)	11 (0.2)	9 (0.2)	< 5 (0.2)	5 (0.1)	12 (0.3)	6 (0.5)	6 (0.2)
Other mental and behavioural disorders (F10.8)	28 (0.3)	18 (0.5)	10 (0.2)	17 (0.3)	10 (0.5)	7 (0.2)	11 (0.3)	8 (0.6)	< 5 (0.1)
Unspecified mental and behavioural disorders (F10.9)	67 (0.7)	25 (0.7)	42 (0.7)	42 (0.7)	16 (0.7)	26 (0.7)	25 (0.6)	9 (0.7)	16 (0.6)
Age at admission	**Mean (Median) Age at admission**								
Clinical condition not specified (F10)	43.96 (44)	42.63 (42)	44.77 (44)	42.55 (42)	41.43 (41)	43.26 (42)	46.59 (46)	45 (45)	47.52 (47)
Acute intoxication (F10.0)	44.39 (44)	40.14 (39)	46.59 (46)	42.95 (42)	38.7 (37)	45.52 (44)	47.26 (48)	44.15 (45)	48.42 (51)
Harmful use (F10.1)	42.63 (42)	40.16 (38)	44.34 (44)	40.4 (39)	38.44 (37)	41.9 (42)	46.45 (47)	43.66 (42)	48.04 (48)
Dependence syndrome (F10.2)	49.93 (50)	48.16 (48)	50.82 (51)	48.03 (47)	46.84 (46)	48.7 (48)	52.15 (53)	49.93 (50)	53.17 (54)
Withdrawal state (F10.3)	50.09 (49)	46.28 (45)	52.63 (51)	48.66 (46)	45.19 (44)	51.39 (51)	52.15 (51)	48.29 (49)	54.15 (54)
Withdrawal state with delirium (F10.4)	49.82 (50)	45.56 (45)	53.82 (57)	48.59 (51)	43.64 (43)	53.55 (58)	52.27 (49)	49.8 (46)	54.33 (50.5)
Psychotic disorder (F10.5)	40.81 (39)	40.17 (37)	41.29 (41)	39.49 (37.5)	38.89 (35)	39.97 (39)	43.83 (42.5)	43.3 (41)	44.21 (45)
Amnesic syndrome (F10.6)	66.74 (68)	72.91 (73)	63.92 (64)	64.27 (65.5)	67.75 (66.5)	63.64 (62.5)	73.89 (73)	75.86 (77)	67 (67)
Residual and late onset psychotic disorder (F10.7)	55.43 (62)	58.6 (65)	52.55 (55)	53.22 (54)	55.75 (61.5)	51.2 (54)	57.08 (62.5)	60.5 (64.5)	53.67 (58.5)
Other mental and behavioural disorders (F10.8)	41.32 (41)	41.5 (43)	41 (39)	43.71 (44)	43.4 (44.5)	44.14 (43)	37.64 (40)	39.13 (40)	33.67 (32)
Unspecified mental and behavioural disorders (F10.9)	46.57 (44)	45.32 (38)	47.31 (46)	45.81 (42)	43.63 (37.5)	47.15 (50.5)	47.84 (46)	48.33 (48)	47.56 (44)
Total	**46.88 (46)**	**44.91 (44)**	**48 (48)**	**44.94 (44)**	**43.28 (43)**	**45.96 (45)**	**49.77 (50)**	**47.65 (48)**	**50.85 (51)**

### Trend analysis of first, and readmissions for alcohol-related psychiatric hospital admissions, 2015–2024

Between 2015 and 2024, annual admissions for all alcohol-related disorders have declined by 41.4%, from 1,188 in 2015 to 696 in 2024. Specifically, a 12.1% decline was noted between 2019 and 2020, with an additional 20.9% decline from 2020 to 2021, demonstrating the impact of the COVID-19 pandemic national lockdowns.

**Fig. 1:**
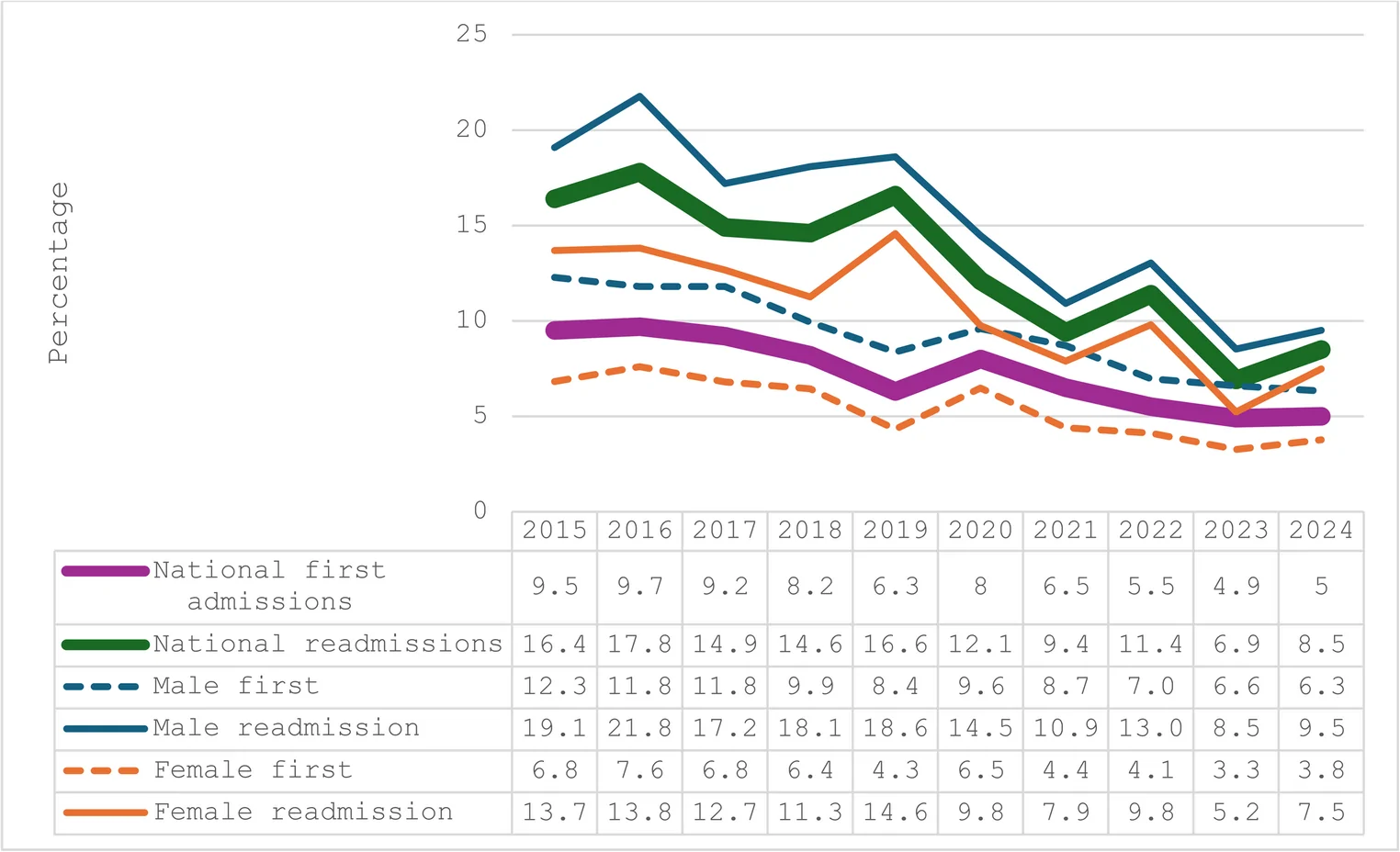
Rate of first admissions and readmissions, for alcohol-related diagnoses, as a percentage of all admissions, by sex 2015–2024

As with overall alcohol-related disorder admissions, when examined by order of admission, rates per 100,000 of the population for both first admissions and readmissions declined during the study period (Fig. 1). Rates of first admissions declined by 47.4% between 2015 and 2024 with a slightly higher declination of rates for readmissions noted (48.2%) for the same period. First admissions increased between 2019 and 2020, likely due to the recommendation in the 2020 mental health strategy whereby those with coexisting mental health problems and substance dependency could be treated in psychiatric units [10].

## Discussion

There were 9,586 cases of admissions for alcohol-related disorders in psychiatric units in Ireland between 2015 and 2024, representing 5.9% of all psychiatric admissions. Nearly two-thirds (63.8%) were readmissions, placing alcohol-related disorders among the top five diagnostic categories for repeat psychiatric hospitalisation, alongside personality, bipolar, and psychotic disorders [22].

Readmissions for psychiatric conditions are well-documented internationally, ranging from 9% to 84% [15] with alcohol-related disorders a consistent predictor [13, 26–28]. A previous study in Ireland found that one-fifth of readmissions were for alcohol-related disorders [16]. The authors of that study questioned the rehabilitative value of short inpatient stays. Our findings that half (49.8%) of all readmissions for alcohol-related disorders had dependence syndrome supports concerns about limited long-term benefits of repeated hospitalisations. While just 2.5% of overall bed days in psychiatric units and hospitals in 2024 were accounted for by alcohol-related disorders, 69.6% of bed days were readmissions, costing an estimated EUR 8.6 million annually [22, 29].

These figures highlight the need for more targeted treatment pathways and a dedicated alcohol clinical programme, similar to those established for other complex disorders [30]. Reliable data from community mental health services and a unique health identifier are essential to evaluate the effectiveness of treatments. In the absence of such treatment evaluations and the wider issue of poor community services data, inpatient data shows repeated inpatient care continues to incur substantial societal and health care costs. Strengthening community-based, integrated alcohol services could reap huge benefits.

As alcohol remains the most commonly used and harmful substance in Ireland, fully implementing and enforcing all elements of the Public Health (Alcohol) Act 2018 and carefully considering the objectives of national drug and alcohol strategy remain crucial [31–34].

The 2020 mental health strategy highlights the issue of dual diagnosis (mental health conditions along with substance use disorders) promoting rehabilitation and recovery and strategic action plan number 2.1.24 explicitly calls to improve outcomes for this population. With the new drugs and alcohol strategy currently being considered, the evidence provided in this study highlights the urgency of strengthening community supports and services and expanding integrated alcohol services for a streamlined referral process [35].

Involving people with lived experience of mental health and alcohol-related disorders in policymaking offers valuable insights into reducing repeat admissions. A key project within the VISTA (VISion To Action) research programme, funded by the HRB, focuses on enhancing Public and Patient Involvement (PPI) in mental health research [36]. The research recommendations will help inform future initiatives aimed at reducing readmissions.

Readmissions in our study aligned with existing literature and were most common among employed, single men, aged 45–64 years with alcohol dependence. Although ‘revolving door’ admissions are defined differently across the literature, previous studies have shown a stronger association between younger age, low education, and unemployment as being positively associated with readmissions [13, 20, 21, 37]. While previous research has linked low socioeconomic status, including unemployment and lower levels of education with higher rates of readmissions to psychiatric services, our findings indicate that individuals who are employed, those in non-manual positions, and those admitted to private hospitals or units (potentially reflecting higher socioeconomic status) may be more likely to be re-admitted [13]. However, our findings of male sex and single marital status align closely with much of the literature in this area [12, 13, 17–19, 38, 39]. Noteworthy are the number, albeit small number, of admissions among those aged less than 25 years.

Women in our cohort were older than men and less likely to self-refer for treatment, reflecting a potential reluctance among women in Ireland to engage with drug and alcohol treatment services and psychiatric services due to stigma, fear, and shame [40, 41]. One of the main factors affecting engagement with services has been noted to be fear of losing their children through the involvement of social services, with lack of childcare facilities and previous negative experiences with service providers also expressed [40, 41]. Late referrals to specialised treatment for women by health services coupled with perceived barriers to assessing treatment or interventions at an earlier stage suggest treatment services are failing to reach a vulnerable cohort of people. These findings highlight the need for sex-specific, stigma-free treatment models, a priority under Ireland’s national strategy [34].

The key objectives of ‘The European Mental Health Action Plan’, such as ensuring mental health services are accessible and affordable, and provide respectful, safe, and effective treatment, should be resourced [42]. However, mental illness-related stigma remains a barrier to accessing effective treatment [43]. Evidence from a systematic review and meta-analysis suggest all types of contact and education interventions are effective in reducing stigma towards people with mental illness in the short-term and should be implemented more widely [44].

Our findings point to a higher per population rate of alcohol-related psychiatric admissions in rural areas, consistent with findings reported in an earlier study conducted in 1985 [45] and also aligning with previous Irish research [46–48]. However, more up-to-date and international literature results in mixed evidence, with urban areas commonly associated with higher risk [49, 50], or no significant urban-rural association [51–53].

Understanding factors associated with readmissions may help practitioners identify those most at risk for readmission and work towards identifying their particular needs to reduce their burden on already stretched psychiatric services in Ireland. Among alcohol-related psychiatric admissions, almost two-thirds are re-admitted to psychiatric units. Efforts to identify and improve interventions to address their needs at first admission are warranted as well as considering alternative treatment pathways to inpatient treatment. It should be noted that alcohol-related readmissions were more likely to involve stays of under one week. Given the single marital status, yet employed status of readmissions in our sample, social isolation may be a factor and those admitted for alcohol-related disorders may benefit from psychosocial interventions available in psychiatric outpatient services, given the possible lack of familial support.

The findings from this study reinforce the call for brief interventions at every opportunity for those with harmful, or potentially harmful alcohol use [54, 55].

### Strengths and limitations

This is the first study in Ireland to examine the revolving door phenomenon in alcohol-related admissions using NPIRS data. However, the lack of a national unique health identifier in the data during the study period makes it difficult to look at individual service use and treatment. It is not possible to follow the patient journey within the inpatient services or indeed from community services to inpatient services. There is no way of knowing if an individual patient has attended several units and hospitals with alcohol-related disorders over the course of a particular year or if they have previously attended community services. The introduction of the Individual Health Identifier (IHI) and the use of the Personal Public Service Number (PPSN) as a unique identifier as proposed in the upcoming Health Information Bill will hopefully allow such analysis in the future.

A relatively large proportion of demographic information is ‘unspecified’ or ‘unknown’ which may result in bias in the sample. Further information about living arrangements (i.e. who the patient lives with) and the extent of social supports would be beneficial. Additional insights into the demographic variables would enable greater characterisation of readmissions.

While readmissions captured in this study relate to readmissions with a diagnosis of alcohol-related disorders, it is not known what diagnosis the patient had on any previous inpatient admission or indeed on their first admission. It may have been for a psychiatric diagnosis unrelated to alcohol-related disorders so it should not be assumed that any previous diagnosis was an alcohol-related one.

It should also be noted that NPIRS captures up to three additional diagnoses but for this study, we only examined those with a primary diagnosis of an alcohol-related disorder. Therefore, the number with an alcohol-related diagnosis is likely much higher. This also means that potentially the second or subsequent diagnosis could have impacted the length of stay. It is also unknown the intervening period between first, and readmissions, and how many times the same patient is re-admitted and if they were re-admitted for the same diagnosis. It is known through previous evidence, that non-compliance with treatment and those with a dual diagnosis are more likely to be re-admitted but our data would not allow this analysis. This information would allow greater understanding of the main features that are associated with readmissions.

## Conclusions

This study aimed to investigate the characteristics of alcohol-related admissions to psychiatric inpatient services in Ireland, with a specific focus on differences between first and repeat admissions, during the period 2015–2024 inclusive. This is the first time that repeat admissions for alcohol-related diagnoses have been examined in Ireland. Alcohol-related admissions account for approximately 6% of all admissions, and although representing a relatively small share, considering the proportion that are repeat admissions annually and the accumulated annual beds days, represent a substantial burden on psychiatric services. This study also highlights important sex differences with women more likely to be older presenting for psychiatric treatment, potentially due to the stigma associated with seeking support for their alcohol use. This study also encourages a wider discourse about urban-rural differences in alcohol-related psychiatric admissions. Although beyond the scope of our study, further scrutiny of our finding that there was greater representation of rural areas may provide valuable insights.

This analysis provides an important insight into this cohort and can be used to build on strategies to reduce readmissions and steer potential readmissions towards effective community-based services.

## Supplementary Information

Below is the link to the electronic supplementary material.

Supplementary Material 1 (DOCX 46.5 KB)

## Data Availability

Requests for data from NPIRS will be considered and may be made by contacting npirs@hrb.ie.
