# Supplementary material for: Alcohol-related psychiatric inpatient admissions in Ireland – characteristics, trends and factors associated with first and repeat admissions, 2015–2024
Source: Ir J Med Sci. 2026 Jun 6;195(5):2963–74. doi: 10.1007/s11845-026-04420-0 (PMC13740114; doi:10.1007/s11845-026-04420-0)
Supplement: Supplementary file 1 [file 11845_2026_4420_MOESM1_ESM.docx]

## Supplementary tables

Supplementary Table 1 Mean (median) age of all alcohol-related admissions by urban-rural location, 2015 – 2024

|  | Urban | Rural | Total |
| --- | --- | --- | --- |
|  | Mean (median) | | |
| Clinical condition not specified | 45.63 (45) | 41.85 (41) | 43.94 (43) |
| Acute intoxication | 44.3 (44) | 44.48 (44) | 44.38 (44) |
| Harmful use | 42.89 (41) | 42.16 (43) | 42.63 (42) |
| Dependence syndrome | 49.74 (49) | 50.56 (51) | 49.93 (50) |
| Withdrawal state | 50.09 (48) | 50.11 (49) | 50.09 (49) |
| Withdrawal state with delirium | 48.63 (46) | 51.43 (56) | 49.82 (50) |
| Psychotic disorder | 42.1 (41) | 36.8 (32) | 40.73 (39) |
| Amnesic syndrome | 65.37 (68) | 69.73 (73) | 66.74 (68) |
| Residual and late onset psychotic disorder | 54.23 (54) | 57.38 (65) | 55.43 (62) |
| Other mental and behavioural disorders | 43.24 (40) | 37.7 (36) | 41.19 (40) |
| Unspecified mental and behavioural disorders | 48.61 (48) | 40.06 (42) | 46.57 (44) |
| **Total** | **47.71 (47)** | **45.17 (45)** | **46.88 (46)** |
